# SGLT2 inhibitors and bone health in CKD

**DOI:** 10.1093/ckj/sfag021

**Published:** 2026-02-11

**Authors:** Mathias Haarhaus

**Affiliations:** Karolinska Institutet, Department of Clinical Science, Intervention and Technology, Division of Renal Medicine, Karolinska University Hospital, Stockholm, Sweden; Diaverum Sweden, Malmö, Sweden

Chronic kidney disease (CKD) poses a major threat to global health. With a prevalence of >10%, CKD is estimated to be among the leading causes of premature death. The introduction of sodium glucose cotransporter 2 inhibitors (SGLT2i) has had a considerable impact on disease progression, life-expectancy, and quality of life of patients with CKD, independent of underlying cause [1]. Consequently, screening programs for CKD in the general population have become cost-effective [2]. While initially developed for the treatment of diabetes mellitus type 2 (T2DM), clinical trials in CKD patients have demonstrated impressive effectiveness of SGLT2i to prevent CKD progression, cardiovascular complications, and premature death, independent of DM status or the presence of proteinuria. SGLT2i are generally well tolerated. Adverse effects reported in clinical trials and post-market surveillance include urinary tract infections, dehydration, and diabetic ketoacidosis. Early observations of an increased fracture risk and decreased bone mineral density (BMD) were alarming but could not be reproduced consistently in later studies.

Fracture incidence is increased in all stages of CKD, resulting in reduced quality of life, loss of patient autonomy, and increased mortality. The Kidney Disease: Improving Global Outcomes (KDIGO) initiative has recently introduced the term CKD-associated osteoporosis, which summarizes the underlying processes, combining CKD-specific disturbances of bone turnover, mineralization, volume, and material properties with established causes of primary and secondary osteoporosis [3]. This new definition opens for novel therapeutic strategies to reduce fracture risk in CKD, including the use of interventions targeting bone directly. It will also improve the understanding of the clinical consequences of interventions that affect mineral metabolism in CKD.

## WHAT IS THE MECHANISM OF SODIUM GLUCOSE COTRANSPORTER 2 INHIBITORS (SGLT2i) ON MINERAL AND BONE METABOLISM?

SGLT2 is primarily expressed in renal tubular cells, whereas it does not seem to be expressed in bone. SGLT2i exert their main therapeutic effect by inhibiting the sodium and glucose re-uptake in the proximal renal tubule, increasing sodium and glucose excretion and thereby improving glycemic and blood pressure control. The resulting increase in tubular sodium concentration stimulates phosphate reabsorption by proximal tubular sodium-phosphate counter-transport. In healthy volunteers, canagliflozin induces a rapid increase of serum phosphate, paralleled by an increase in circulating levels of the phosphatonins fibroblast growth factor 23 (FGF23), and parathyroid hormone (PTH), and a decrease of 1,25 (OH)_2_ vitamin D [4]. A similar effect has been described for dapagliflozin in T2DM patients. FGF23 and PTH increase phosphaturia by inhibiting the tubular reabsorption of phosphate. Furthermore, FGF23 is a strong inhibitor of vitamin D activation in the kidneys and may inhibit bone mineralization, whereas PTH stimulates bone turnover. Most studies evaluating the effect of SGLT2i on bone metabolism are limited by small sample size, short follow-up time, and a variation of comparator treatments. However, a recent meta-analysis, including 25 publications comprising 22 828 study participants with follow-up periods between 12 and 104 weeks, demonstrated an SGLT2i treatment induced increase in circulating PTH and the bone resorption marker Collagen I C-terminal telopeptide (CTX), paralleled by a decrease in alkaline phosphatase (ALP), which reflects bone formation in the absence of liver disease [5]. This possible effect of SGLT2i on bone metabolism is further supported by a mendelian randomization study, based on a large GWAS database. Inhibitory SGLT2 mutations were associated with increased circulating levels of phosphate, FGF23, and the bone resorption marker tartrate-resistant acid phosphatase 5b (TRACP5b) and with decreased levels of ALP [6].

## WHAT ARE THE CLINICAL CONSEQUENCES OF THE EFFECT OF SGLT2i ON MINERAL AND BONE METABOLISM IN CKD?

The CANVAS study demonstrated an increased fracture risk with canagliflozin compared to placebo in T2DM patients without CKD [7]. While other large clinical trials with canagliflozin or other SGLT2i could not replicate these findings, a minor trial including T2DM patients with CKD stage 3 found a significantly increased fracture risk in the dapagliflozin arm, compared to no fractures in the placebo arm [8]. However, none of the large clinical trials were designed to detect a difference in fractures, and minor studies are flawed with a lack of power to predict fracture outcomes. In a large population-based observational study, SGLT2i treatment was associated with increased fracture risk compared to other oral anti-diabetic drugs only in patients with the highest accumulated dose, indicating that treatment time may be an important factor [9]. In addition, some studies demonstrated a decrease in BMD with SGLT2i treatment while others could not detect any effect, but again, study size and design were not optimized for BMD outcomes. Besides insufficient power and non-skeletal primary endpoints, differences in capturing fractures may contribute to the divergent results in single studies and meta-analyses evaluating the skeletal safety of SGLT2i. Furthermore, an increased risk of falls in patients on SGLT2i treatment, which has been attributed to a blood pressure-lowering effect and an increased incidence of diabetic ketoacidosis, may contribute indirectly to an increased fracture risk. Remarkably, evidence for skeletal effects from SGLT2i in patients with eGFR <30 ml/min/1.73 m^2^ is non-existent, although these drugs are not contraindicated in advanced CKD, where bone abnormalities and fracture risk are highly increased. Adequately powered studies with relevant bone-related endpoints are needed to address the skeletal safety of SGLT2i in CKD.

A recent report by Elshayeb *et al.* [10] addresses the important question of the skeletal impact of SGLT2i in non-diabetic patients with CKD stages 2–4. They found no difference in the effect of 1 year of treatment with dapagliflozin 10 mg vs. placebo on changes in CTX or volumetric BMD of the spine. These seemingly reassuring results should be interpreted with some caution, as the power analysis was based on CTX results from a study involving non-CKD patients. CTX demonstrates a considerable variability in CKD due to renal clearance, a large diurnal variation, and the increased spectrum of bone turnover disturbances in CKD. Results from studies in non-CKD patients are of limited value for power calculations in CKD as they may underestimate the required study size. Another bone turnover marker (BTM) determined in the study, total pro-collagen I N-terminal propeptide (PINP), is also influenced by kidney function and should not be used in studies including patients with advanced CKD. In addition, only rudimentary information on concomitant medication with potential to influence results is reported and BMD was only determined in the lumbar spine, whereas the previously described impact of SGLT2i on BMD was restricted to the total hip. While the authors acknowledge some of these limitations, the risk of overinterpretation of this potentially underpowered study remains. Even more concerning is the risk of amplifying biases by combining several inadequately designed studies in meta-analyses and systematic reviews.

## WHAT ARE ADEQUATE INDICATORS OF BONE HEALTH IN CKD?

Cardiovascular disease, fractures, and all-cause mortality are well-established complications of CKD-associated mineral and bone disorder (CKD-MBD) and can be linked to impaired renal handling of minerals, resulting in a complex disorder, including phosphate retention, increased levels of phosphatonins, reduction of klotho expression, impaired vitamin D activation, vascular calcification, and disturbed bone turnover. Circulating concentrations of calcium, phosphate, and the mineral regulator PTH are recommended treatment targets in CKD-MBD with clear definition of target ranges. However, these target ranges are largely based on observational data, and target achievement by therapeutic interventions is not consistently liked to better outcomes. Association of these biomarkers with skeletal outcomes are inconsistent in CKD, and more direct indicators of bone health, e.g. BMD or BTM, may be better suited as predictors of fracture risk and as treatment targets for fracture prevention strategies in CKD [3].

Several clinical studies have established associations between BMD and the incidence of fractures in CKD, including CKD G5D and post-transplant CKD. Interventions targeting bone mass demonstrate often greater treatment effects in CKD, depending on the greater bone turnover abnormalities in these patients. This applies to the use of antiresorptive treatment in increased bone turnover states and the use of anabolic agents in reduced turnover states. The clinical relevance of BMD and its modifiability in CKD renders it a meaningful target for clinical management and interventional studies in this population.

Disturbances of bone turnover are a key feature of the disturbed mineral balance in CKD. A bone biopsy with histomorphometric analysis is required for the exact diagnosis of bone turnover disturbances, but it is not a suitable procedure for large-scale trials or clinical management of patients with CKD, since the procedure is invasive and the number of laboratories performing the histomorphometric method is highly limited. Instead, current clinical guidance relies largely on PTH for the management of bone turnover abnormalities in CKD, ignoring the fact that it does not directly reflect bone turnover and that interventions targeting bone directly, e.g. antiresorptive treatment, may cause a dissociation of PTH and bone turnover. Circulating BTM reflect bone turnover with considerable accuracy, outperforming PTH in most diagnostic studies. Thus, a bone biopsy may not be necessary in most clinical settings where knowledge of bone turnover is of interest. Importantly, BTM are suited for longitudinal evaluation and reflect bone turnover also in patients treated with direct bone-targeting agents, thus overcoming the limitations of bone biopsies and PTH as clinically useful biomarkers. Furthermore, BTM can predict CV events and fractures in CKD and may even outperform BMD for fracture risk prediction in advanced CKD.

Several BTM are renally excreted and thus of limited value in CKD. This applies to CTX, the reference BTM for bone resorption, and to total PINP, the reference BTM for bone formation, widely used for management of patients with preserved kidney function. Therefore, a recent international consensus initiative has defined the bone formation marker bone-specific ALP and the bone resorption marker TRACP5b as reference BTM specifically for use in patients with CKD stages 4–5D [11]. These markers are better suited than PTH, calcium, and phosphate to the routine clinical management of bone disease in CKD and should be included in all clinical studies of interventions targeting the skeleton in these patients. Owing to the progressive character of CKD, the use of the CKD-specific reference BTM may be meaningful already in earlier stages of CKD to guarantee continuity.

## CONCLUSIONS

The introduction of SGLT2i has had a considerable impact on the management of patients with CKD. Consequently, their use increases in all stages of the disease. A possible negative effect on bone, paired with an increased fracture risk, is a caveat, especially in patients with advanced CKD, where fracture risk is already increased. Extrapolating evidence from non-CKD populations may be inadequate due to the complexity of mineral disturbances in CKD. In addition, the non-invasive evaluation of bone metabolism in advanced CKD requires the use of non-renally cleared BTM. There is an imminent need for adequately powered studies of skeletal effects of SGLT2i in advanced CKD, ideally using fracture risk as endpoint, alternatively targeting BMD or the reference BTM in CKD, bone-specific ALP, and TRACP5b, as surrogate endpoints.
